# GP utilisation by education level among adults with COPD or asthma: a cross-sectional register-based study

**DOI:** 10.1038/npjpcrm.2016.27

**Published:** 2016-06-09

**Authors:** Øystein Hetlevik, Hasse Melbye, Sturla Gjesdal

**Affiliations:** 1 Department of Global Public Health and Primary Care, University of Bergen, Bergen, Norway; 2 General Practice Research Unit in Tromsø, The Arctic University of Norway, Tromsø, Norway

## Abstract

There is a marked socioeconomic gradient in the prevalence of chronic obstructive pulmonary disease (COPD) and asthma, but a large proportion of patients remain undiagnosed. It is a challenge for general practitioners (GPs) to both identify patients and contribute to equity and high quality in services delivered. The aim of this study was to identify patients with COPD and asthma diagnoses recorded by GPs and explore their utilisation of GP services by education level. This was a cross-sectional, national, register-based study from Norwegian general practice in the period 2009–2011. Based on claims from GPs, the number of patients aged ⩾40 years with a diagnosis of COPD or asthma and their GP services utilisation were estimated and linked to the national education database. Multivariate Poisson and logistic regression models were used to explore the variations in GP utilisation. In the population aged ⩾40 years, 2.8% had COPD and 3.8% had asthma according to GPs’ diagnoses. COPD was four times more prevalent in patients with basic education than higher education; this increase was ⩽80% for asthma. Consultation rates were 12% higher (*P*<0.001) for COPD and 25% higher (*P*<0.001) for asthma in patients with low versus high education in the age group of 40–59 years after adjusting for comorbidity, and patient and GP characteristics. Approximately 25% of COPD patients and 20% of asthma patients had ⩾1 spirometry test in general practice in 2011, with no significant education differences in adjusted models. The higher consultation rate in lower-education groups indicates that GPs contribute to fair distribution of healthcare.

## Introduction

Epidemiological studies across Europe indicate a prevalence of chronic obstructive pulmonary disease (COPD) among adults of up to 10%, as defined by reduced lung function.^1,2^ A recent population study in Norway showed a prevalence of 7.1% for moderate disease, as defined by Global Initiative for Chronic Obstructive Lung Disease (GOLD) stage II, and 1.2% for severe disease (GOLD stage III–IV).^2^ However, the prevalence of COPD diagnoses according to medical records is lower, and it indicates that two in three people with COPD may be undiagnosed.^3,4^ The prevalence of asthma is estimated at 4–7% in people aged ⩾45 years.^5^ COPD and asthma are partly overlapping diseases,^6,7^ and differentiating between them is sometimes difficult in general practice.^8–10^

There appears to be a socioeconomic gradient in the prevalence of COPD and asthma.^11–17^ A Norwegian population-based study found a fourfold increase in obstructive lung disease among people aged >40 years with primary education only compared with those with college education or higher, after adjusting for smoking habits and occupational exposures.^13^ Lower socioeconomic status (SES) has also been found to be associated with reduced health-care access, infrequent monitoring with spirometry and worsening of the prognosis of COPD.^11,18–20^

In general, the rate of general practitioner (GP) consultations in Norway is higher among patients in lower socioeconomic groups, whereas this association is reversed in specialised care.^21^ To our knowledge, there are no studies on variation in GP utilisation related to SES among patients with asthma or COPD. Such variations can be important in diseases with marked SES gradients.

In many countries, GPs have the main responsibility to diagnose and follow-up patients with mild-to-moderate grades of COPD or asthma,^22,23^ involving the specialist only in severe disease. Increasing use and improving skills in the use of spirometry have been shown to identify more patients with COPD than symptom assessment alone and may lead to improved treatment.^24,25^ Current guidelines for COPD and asthma care include regular use of spirometry in general practice.^22,23,26^ In a Norwegian study from 2007, 91% of GP practices reported that they had a spirometer, and that the test was performed by practice assistants in 66% of cases.^27^

Patients with obstructive lung disease often also need health services for comorbid conditions that have an important impact on function and quality of life.^28,29^ From this perspective, an assessment of GPs’ total care for patients with COPD and asthma may reveal useful information for health-care development.

The aims of this study were first to estimate possible differences in the prevalence of COPD and asthma diagnoses recorded by GPs in adults by education level, and second to assess the variation in utilisation of GP services in patients with such diseases according to education level and other GP and patient characteristics, including comorbidity.

## Results

**Figure 1:**
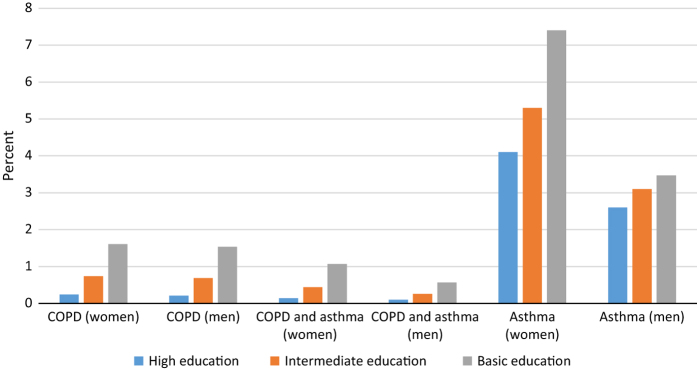
Prevalence of COPD without asthma, both COPD and asthma, and asthma without COPD according to diagnoses used in Norwegian general practice during 2009–2011 among patients aged 40–59 years by gender and education level.

**Figure 2:**
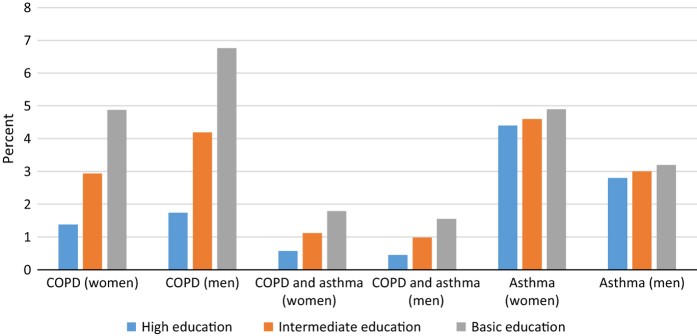
Prevalence of COPD without asthma, both COPD and asthma, and asthma without COPD according to diagnoses used in Norwegian general practice during 2009–2011 among patients aged ⩾60 years by gender and education level.

In Norway, during 2009–2011, according to GP claims, 67,832 people (2.8%) received a COPD diagnosis and 109,771 (3.8%) received an asthma diagnosis in the total population aged ⩾40 years. As 17,297 of these patients were given both a COPD and an asthma diagnosis by the GP during the 3-year period, the total number of patients diagnosed with obstructive pulmonary disease were 160,306 (6.7%). Figures 1 and 2 show the prevalence of obstructive lung diseases recorded by Norwegian GPs by gender, age and educational level.

Among patients with COPD, 44% had only basic education, 47% had intermediate education and 7% had higher education, and for 2% the data were missing. The respective figures for patients with asthma were 27, 49 and 20%, and 4% missing.

### Comorbidity

GP diagnoses collected during 2009–2011 showed a high frequency of comorbid conditions among patients with obstructive lung diseases (Table 1), increasing with lower level of education. The prevalence of depression and anxiety, recorded by GPs, decreased with age, whereas the somatic conditions increased with age.

Among COPD patients aged 40–59 years, 26.5% in the highest and 35.1% in the lowest education group had a consultation with a psychological main diagnosis in 2011, compared with 18.4 and 27.7% among patients with asthma (not tabulated). These percentages were lower and showed less variation among patients aged ⩾60 years.

### GP service utilisation

Table 2 shows a gradual decrease in consultation rates with increasing education level. More than 49% of patients among all COPD patients had five consultations or more.

COPD was used as the main diagnosis by the GP in 22.0% (*N*=97,825) of all consultations among patients in the COPD group, and asthma was the main diagnosis in 14.4% (*N*=72,118) of consultations among patients with asthma.

Approximately 25% of COPD patients and 20% of asthma patients had at least one spirometry test in general practice in 2011.

When comparing the quartiles of GP group practices according to general use of spirometry, the annual rate per 100 list patients was <1 in the lowest quartile, 1–2 in the second, 2–3.6 in the third and >3.6 in the quartile with most frequent use of spirometry.

### Predictors for higher GP consultation rates

In unadjusted analyses (not shown in tables) using education level as a predictor for consultation rates with high education as reference, the COPD patients with basic education only had an incident risk ratio of 1.21 (95% CI=1.14–1.28) in the age group of 40–59 years and 1.06 (1.03–1.10) in the age group ⩾60 years. The incident risk ratios among asthma patients was 1.40 (1.36–1.43) and 1.18 (1.14–1.21), respectively, for these age groups. The effect of education was reduced, but it was still significant in a multivariate model except in COPD patients aged ⩾60 years (Table 3). The presence of a comorbid condition was significantly associated with increased consultation rates in patients with any obstructive lung disease in both age groups (Table 3).

### Predictors for the use of spirometry

There was a slight increase in spirometry rate with higher education in unadjusted analyses. However, multivariate analyses showed no association between education level and the use of spirometry in any of the age or diagnosis groups in multivariate models (Table 4). Stratified analyses revealed no gender differences (not tabled).

However, the rate of spirometry tests in the practice population in the group practice that the regular GP belonged to had a significant impact, increasing the odds for spirometry six to nine times when comparing offices in the lower with the upper quartile.

## Discussion

### Main findings

The present register study based on the data from all Norwegian GPs showed a prevalence of COPD of 2.8% and 3.8% for asthma recorded by GPs in the population aged ⩾40 years. For COPD, the prevalence as recorded by GPs was four times higher in low compared with high education groups. Among women aged 40–59 years, the prevalence of asthma diagnosis in GP medical records increased from 4.1 to 7.4% when comparing higher with low education groups, with markedly less variation in the other age and gender groups.

The GP consultation rate was higher in lower education groups, even after adjusting for comorbid conditions, whereas the use of spirometry as an indicator of quality of care was not associated with patients’ education level.

### Interpretation of findings in relation to previously published work

Compared with a Norwegian population-based study showing a prevalence of COPD GOLD II–IV of 8.3%,^2^ the present finding of a 2.8% prevalence of COPD diagnoses recorded by GPs support earlier findings of a large discrepancy between prevalence estimates based on medical records and population-based screening studies.^3,4^ The frequency of diagnosed COPD or asthma found in GP medical records should not be used as prevalence estimates at the population level, as a large proportion of these patients either do not seek medical treatment or the disease is not recognised by the physicians.

The prevalence of all GP-diagnosed obstructive lung diseases in the age group >40 years is slightly higher than that found in a Dutch study from general practice reporting 5.2%.^30^ However, the prevalence of COPD diagnosis recorded by GPs of 2.8% in this study is lower than the 5.3% found in a Canadian study also using the data from electronic records.^31^

Among COPD patients in the present study, 25.5% also had an asthma diagnosis registered, in line with a recent systematic review showing that patients with both diagnoses constitute between 15 and 55% of COPD patients, depending on the study sample.^7^ However, the proportion of patients with COPD who also had an asthma diagnosis registered was markedly lower than the 55% found in a previous Norwegian study from primary care,^10^ but they registered diagnoses over a 5-year time span.

The fourfold increase in the prevalence of COPD diagnoses recorded by GPs from high to low education level is consistent with other studies, but the smaller SES gradient seen among asthma patients was in contrast to some previous studies.^13,15^

In the present study, health-care access indicated by GP consultation rates corresponded with a low education profile, whereas education was not associated with the use of spirometry. This contrasts with a UK study showing lower use of spirometry among socioeconomically deprived patients,^18^ and in part with a Danish study that found lower use of spirometry among patients with the highest level of education.^19^

Patient education and treatment plans are important elements in care for patients with obstructive lung diseases, and low health literacy may reduce outcome.^32^ Health literacy is related to educational level,^33^ and higher consultation rates for patients in lower educational groups may indicate that GPs to some extent address the increased need for patient education in this group.

Depression and anxiety have negative consequences on the somatic morbidity and quality of life in patients with COPD,^34^ and earlier findings have shown substantial psychosocial burden related to COPD.^28^ We found that one-third of COPD patients had a GP consultation in 2011 with a psychological diagnosis and 19–24% of the patients aged 40–59 years with COPD received a GP diagnosis of depression during the previous 3 years. This indicates that Norwegian GPs address mental health problems to a considerable extent in their consultations with patients with asthma or COPD.

### Strengths and limitations of this study

This was a nationwide study and included claims from all regular GPs in Norway, thus avoiding the selection bias that is common in studies recruiting GPs who wish to participate in research. We used complete data on GP utilisation from 1 year (2011), including all consultations with the GPs in the national list patient systems, covering 99.5% of the population.

One major limitation is that 90% of claims reported to the Norwegian Health Economics Administration (HELFO) include only one International Classification of Primary Health Care code, even if the GP deals with several issues in the consultation. This may result in underestimations of the number of patients diagnosed with COPD and asthma by the GPs. When patients come for other conditions, the COPD or asthma diagnoses may not be reported even if known by the GP. Correspondingly, there is probably an underreporting of comorbid conditions as well. In 2011, 237,034 people aged ⩾40 years were dispensed bronchodilators in Norway, according to the National Prescription Database^35^, and probably most of these prescriptions were given by GPs. The present study identified 160,306 people with the diagnosis of obstructive lung disease in GP records, and this discrepancy indicates that the diagnosis in claims data underestimates the number of patients with a known diagnosis of asthma or COPD in general practice. However, data from prescription databases are not ‘gold standard’, because some people receive a prescription for cough or dyspnoea without a diagnosis of COPD or asthma, whereas patients with obstructive lung disease sometimes do not use their medication.^36^

There is also uncertainty with the diagnostic precision and use of defined criteria of COPD and asthma in general practice. In a Norwegian study, 68% of GP-registered COPD diagnoses could be verified by spirometry.^10^ A proportion of patients diagnosed with COPD who did not fulfil the diagnostic criteria had asthma and vice versa.^8^ The shift in the use of the diagnosis from asthma to COPD shown in recent decades may still not be complete,^37^ and a proportion of patients diagnosed with asthma may have COPD. Therefore, a strength of this study is that both patients with COPD and asthma diagnoses were included. However, because of this uncertainty of accuracy of the diagnosis in general practice, the estimated frequency of the asthma and COPD overlap syndrome must be used with precaution.

During 2011, spirometry tests were performed in fewer than one-quarter of patients with COPD and asthma, a rate far below the recommendation for annual spirometry.^22^ The present study has no data from secondary care, but some of the patients identified by the GPs are followed up by a pulmonary specialist. From other sources, we know that 0.3% of the population aged >45 years were hospitalised in 2011 with COPD as the main diagnosis, and there were ~23,000 out-patient consultations with COPD-related diagnoses in the same year.^38^ This represents approximately one-quarter of the GP consultations with the COPD diagnosis used as the main reason for contact.

The utilisation of specialist out-patient care is higher among patients in higher, compared with lower, educational groups, and may contribute to the opposite educational difference seen in general practice.^21^

Educational level, shown to predict outcome in patients with COPD,^20^ and seldom changing among individuals above the age of 40 years, was used as the marker of SES in this study. SES markers such as income or occupation could have been used as well, but a common limitation of these markers is the lack of recent data for the oldest part of the population that constitutes a large proportion of the present study population. However, using data on income or occupation among patients below 65 years could have given a broader view on SES variations in GP utilisation.

### Implications for future research, policy and practice

The prevalence of COPD diagnoses recorded by GPs in this study was about one-quarter of estimates found in population-based screening studies.^2^ Reasons for underdiagnoses are diverse, but the lack of symptom presentation is one.^39^ GPs could be more active in case-finding, and they have the potential to increase the use of spirometry among adult patients with respiratory symptoms and smoking history.^40,41^

There is, however, an ongoing discussion as to what level of disease will be associated with improved health as a result of a COPD diagnosis with or without subsequent preventive interventions and medical treatment.^40,41^

### Conclusion

The higher consultation rate for COPD in lower education groups in this study adds to previous evidence that GP services may be a tool for promoting a ‘fair’ distribution of healthcare. Improved care for patients with COPD and asthma, and their comorbidities, should be an important element in a future comprehensive GP service.

## Materials and methods

A register-based, cross-sectional, nationwide study from general practice in Norway in 2009–2011 was conducted based on three national registers.

The KUHR database contains all claims for fee-for-service from regular GPs. For each patient-related contact, the GP sends a claim to the Norwegian Health Economics Administration (HELFO) along with the patient’s personal identity number. The claims are based on the GP tariff including codes for consultation and use of spirometry, and one or more diagnoses according to the International Classification of Primary Health Care.

The Regular GP database has information about all regular GPs contracted to municipalities and to the HELFO, including age and gender of the GPs, their patient list size, information about group practices and the practice municipality. This database also includes the identity of the patients on each list.

Information on education was retrieved from the National Educational Database.

The information from these databases was linked by Statistics Norway based on personal identity numbers and anonymously made available to researchers, with the necessary permission from the Data Inspectorate.

### Study population

All Norwegian residents aged 40 years or older in 2011 and all GPs were included.

### Classification of patient groups

On the basis of the diagnoses used in GP claims from 2009 to 2011, all patients given the diagnosis of COPD (R95) or asthma (R96) were identified and grouped into an asthma group, a COPD group and a third group of patients with both diagnoses, the asthma and COPD overlap syndrome classified as a phenotype of COPD.^7^ This group is presented as a separate group in descriptive figures and tables. However, the patients with both diagnoses were included in the COPD group in regression analyses, because variation of educational level and results from preliminary regression analyses were similar in these groups.

The term ‘prevalence of COPD (or asthma) diagnoses recorded by GPs’ refers to the proportion of registered patients where these diagnoses were recorded in the GPs’ medical records and reported to the HELFO in a claim during 2009–2011.

### Outcome measure

To estimate the variations in care across strata of educational level, consultation rates in 2011 were estimated as a continuous variable. Use of spirometry test in 2011—one or more times versus no tests—was also identified. These outcomes were used in the regression analyses.

### Explanatory variables

The patient characteristics included were age, gender and education grouped into three levels: basic education (<13 years), intermediate education (13–15 years) and high education (⩾16 years).

Comorbid conditions were identified using International Classification of Primary Health Care diagnoses in GP claims from the period 2009 to 2011, and the following conditions were used as explanatory variables: diabetes (T89–90), hypertension (K85–87), cardiovascular disease (K74–76 and K89–91), depression (P76) and anxiety (P74).

The GP characteristics included were age, gender, being approved as specialist in family medicine or not and list size for each GP.

Practice characteristics were list size of the entire group practice and the total number of spirometry tests performed during 2011 based on all claims from all GPs in each group practice. The practices were grouped into quartiles based on the rate of spirometry in the total population listed in the group practice.

### Statistical analysis

One-way analysis of variance and *χ*^2^-test were used to compare the education distribution, comorbidities and GP service utilisation among patients with COPD or asthma.

Poisson regression models with robust estimates of s.d. were used to analyse the association between total number of GP consultations, and patients’ and GPs’ characteristics. Incident risk ratios are presented and can be interpreted as the increase in rate of the outcome (consultation rate), given that all other variables are unchanged. Logistic regression models were used to identify predictors for the use of spirometry. In the regression models, there is no independency because the patients are linked to a regular GP. This was taken into account using the regular GP as clusters in a two-level model in the analyses. A *P-*value <0.05 was considered significant. Stata Statistical Software (Release 13; College Station, TX, USA) was used.

### Approval

The Norwegian Data Inspectorate and the Norwegian Directorate of Health, being responsible for administration of the registers, approved the linking of data registers. All patient data were anonymised.

## Figures and Tables

**Table 1:** Comorbid diagnoses used in GP records among adult patients diagnosed with COPD, both COPD and asthma, and asthma during 2009–2011 by their GP by diagnosis, education level and age group

	*COPD (no asthma)*				*COPD and asthma*				*Asthma (no COPD)*			
	*Educational level*			P*-value*	*Educational level*			P*-value*	*Educational level*			P*-value*
	*Basic*	*Interm.*	*High*		*Basic*	*Interm.*	*High*		*Basic*	*Interm.*	*High*	
*Patients aged 40–59 years*												
*N*	3,697	4,567	875		1,925	2,196	483		11,464	24,065	12,881	
*Proportion with comorbid conditions (%)*												
Cardiovascular disease	11.0	9.3	6.5	<0.001	10.4	7.5	6.8	0.001	4.9	3.8	2.6	<0.001
Hypertension	19.7	21.2	18.5	0.099	20.1	19.9	17.6	0.47	19.1	18.7	14.3	<0.001
Diabetes	9.7	8.0	6.4	0.002	11.3	8.7	7.9	0.007	8.9	6.2	3.7	<0.001
Depression	22.5	19.1	18.4	<0.001	28.0	22.1	20.1	<0.001	20.3	15.0	13.3	<0.001
Anxiety	14.4	8.5	6.6	<0.001	18.7	10.5	6.8	<0.001	12.1	5.5	3.7	<0.001
												
*Patients aged ⩾60 years*												
*N*	18,391	19,341	2,531		5,566	5,763	812		13,816	20,830	5,624	
*Proportion with comorbid conditions (%)*												
Cardiovascular disease	23.0	21.0	10.0	<0.001	22.6	19.4	17.6	<0.001	17.9	14.4	11.1	<0.001
Hypertension	35.6	34.6	31.9	0.001	37.6	38.1	35.5	0.35	45.1	40.9	35.0	<0.001
Diabetes	13.3	11.4	10.4	<0.001	13.9	12.2	10.8	0.005	15.7	11.5	8.0	<0.001
Depression	11.0	10.2	11.3	0.02	13.2	12.1	12.9	0.20	9.9	8.7	9.4	0.001
Anxiety	6.4	4.4	2.9	<0.001	8.6	6.2	4.3	<0.001	5.8	3.6	2.9	<0.001

Abbreviations: COPD, chronic obstructive pulmonary disease; GP, general practitioner; Interm., intermediate.

**Table 2:** Utilisation of GP services in 2011 among adult patients diagnosed with COPD or asthma in Norwegian general practice^a^ by diagnosis, education level and age group

	*COPD^b^*				*Asthma^c^*			
	*Basic education*	*Intermediate education*	*High education*	P*-value*	*Basic education*	*Intermediate education*	*High education*	P*-value*
*Patients aged 40–59 years*								
*N*	5,622	6,763	1,358		11,464	24,065	12,881	
Gender (% male)	44.3	48.6	40.1	<0.001	35.6	41.2	35.0	<0.001
Age (mean)	52.5	53.1	52.2	<0.001	49.3	49.4	48.6	<0.001
Consultation rate (mean)	6.9	6.3	5.7	<0.001	6.0	5.0	4.3	<0.001
Proportion with of patients with:								
⩾1 consultation (%)	95.0	95.2	94.7	0.76	93.4	92.4	91.3	<0.001
⩾5 consultation (%)	56.8	51.3	49.1	<0.001	49.2	42.7	34.6	<0.001
⩾10 consultation (%)	23.3	19.5	16.9	<0.001	18.3	13.2	9.6	<0.001
Proportion of patients with ⩾1 spirometry (%)	28.6	29.4	27.6	0.32	19.1	19.1	18.7	0.53
								
*Patients aged ⩾60 years*								
*N*	23,961	25,104	3,343		13,816	20,830	5,624	
Gender (% male)	44.2	53.7	57.6	<0.001	29.3	37.4	43.2	<0.001
Age (mean)	73.5	71.8	71.4	0.016	72.9	70.0	67.9	<0.001
Consultation rate (mean)	6.8	6.5	6.4	<0.001	6.3	5.8	5.3	<0.001
Proportion of patients with:								
⩾1 consultation (%)	94.2	94.8	95.1	0.011	94.8	95.2	94.7	0.081
⩾5 consultation (%)	56.6	54.2	53.7	<0.001	53.6	49.8	44.7	<0.001
⩾10 consultation (%)	22.8	21.1	20.1	<0.001	19.8	16.8	14.6	<0.001
Proportion of patients with ⩾1 spirometry (%)	25.1	27.8	27.6	<0.001	18.2	20.5	20.8	<0.001

Abbreviations: COPD, chronic obstructive pulmonary disease; GP, general practitioner.

a Patient identified by diagnosis used by GPs in the 3-year period of 2009–2011.

b Including patients with COPD and both COPD and asthma.

c Including patients with asthma, but no COPD diagnosis.

**Table 3:** Associations between numbers of GP consultations in 2011 among patients diagnosed with COPD or asthma in Norwegian general practice, and patient and GP characteristics

	*COPD*^a^ *40–59 years*		*Asthma 40–59 years*		*COPD*^a^ *⩾60 years*		*Asthma ⩾60 years*	
	*IRR*	*95% CI*	*IRR*	*95% CI*	*IRR*	*95% CI*	*IRR*	*95% CI*
*Education level*								
High (reference)	1		1		1		1	
Intermediate	1.08**	1.02–1.14	1.13***	1.11–1.15	1.00	0.97–1.04	1.04**	1.01–1.07
Low	1.12***	1.06–1.18	1.25***	1.22–1.28	1.02	0.99–1.05	1.04**	1.01–1.07

Male patient	0.83***	0.81–0.86	0.77***	0.76–0.79	0.96***	0.95–0.98	0.91***	0.90–0.93
Patient age (years)	0.99***	0.99–0.99	1.00	1.00–1.00	1.01***	1.00–1.01	1.01***	1.01–1.01
								
*Comorbid conditions*								
Cardiovascular diseases	1.30***	1.23–1.38	1.37***	1.31–1.42	1.24***	1.22–1.27	1.29***	1.26–1.32
Hypertension	1.12***	1.08–1.16	1.26***	1.23–1.29	1.06***	1.04–1.08	1.12***	1.10–1.14
Diabetes	1.37***	1.30–1.45	1.48***	1.43–1.52	1.28***	1.25–1.31	1.34***	1.30–1.37
Depression or anxiety	1.54***	1.49–1.60	1.59***	1.56–1.63	1.30***	1.27–1.33	1.36***	1.33–1.40
								
*GP characteristics*								
GP male	1.09***	1.04–1.14	1.06***	1.03–1.08	1.04**	1.02–1.06	1.03*	1.00–1.06
GP age	0.996**	0.994–0.999	0.997***	0.995–0.998	0.996***	0.995–0.997	0.997***	0.995–0.998
GP approved specialist in FM	0.99	0.94–1.04	1.00	0.97–1.03	0.99	0.96–1.03	0.99	0.96–1.02
GP list size (per 100)	1.00	0.99–1.01	1.00	1.00–1.01	1.00	1.00–1.01	1.004*	1.00–1.01

*N*	13,286		47,012		50,651		39,096	

Poisson regression models by diagnosis and age group.

Abbreviations: COPD, chronic obstructive pulmonary disease; CI, confidence interval; FM, familiy medicine; GP, general practitioner; IRR, incident risk ratio.

a Patients with both COPD and asthma diagnoses are included in the COPD group.

*P<0.05, **P<0.01, ***P<0.001.

**Table 4:** Patient and GP characteristics associated with ⩾1 spirometry test performed in Norwegian general practice in 2011 among patients diagnosed with COPD or asthma during 2009–2011

	*COPD*^a^ *40–59 years*		*Asthma 40–59 years*		*COPD*^a^ *⩾60 years*		*Asthma ⩾60 years*	
	*OR*	*95% CI*	*OR*	*95% CI*	*OR*	*95% CI*	*OR*	*95% CI*
*Education level*								
High (reference)	1		1		1		1	
Intermediate	1.07	0.94–1.23	1.03	0.97–1.09	1.00	0.91–1.09	1.04	0.96–1.12
Low	1.05	0.91–1.21	1.05	0.97–1–13	0.95	0.87–1.04	0.99	0.90–1.08

Male patient	1.09*	1.01–1.18	1.04	0.99–1.10	1.10***	1.05–1.15	1.05	0.99–1.11
Patient age (years)	1.00	0.99–1.01	1.00	1.00–1.01	0.97***	0.97–0.98	0.98***	0.98–0.98
								
*Spirometry rate in the practice*^b^								
Lowest quartile	1		1		1		1	
Medium–low	1.99***	1.65–2.42	2.24***	1.93–2.60	2.31***	2.02–2.66	2.03***	1.72–1.39
Medium–high	3.33***	2.76–4.03	3.28***	2.84–3.79	3.69***	3.23–4.22	3.50***	2.98–4.11
Highest quartile	6.51***	5.38–7.88	7.43***	6.39–8.63	7.96***	6.91–9.16	8.79***	7.46–10.37
								
*GP characteristics*								
GP male	1.14*	1.02–1.27	1.08	0.99–1.19	1.11*	1.03–1.20	1.15**	1.04–1.27
GP age	1.00	0.99–1.00	1.00	0.99–1.00	1.00	0.99–1.00	0.995*	0.990–1.00
GP approved specialist in FM	1.03	0.91–1.17	1.05	0.95–1.16	0.99	0.89–1.10	1.02	0.90–1.16
GP list size (per 100)	1.01	1.00–1.03	1.03***	1.01–1.04	0.99**	1.01–1.03	1.04***	1.02–1.06

*N*	13,325		47,166		50,818		39,182	

Logistic regression models by diagnosis and age group.

Abbreviations: COPD, chronic obstructive pulmonary disease; FM, familiy medicine; GP, general practitioner; OR, odds ratio.

a Patients with both COPD and asthma diagnoses are included in the COPD group.

b The rate of spirometry use in the total (group) practice the patient belongs to, divided in quartiles.

*P<0.05, **P<0.01, ***P<0.001.
